# Oncometabolites in urine – a new opportunity for detection and prognosis of the clinical progress of verified prostate cancer-a pilot study

**DOI:** 10.55730/1300-0144.5363

**Published:** 2022-01-23

**Authors:** Dimitar DELKOV, Lyubka YOANIDU, Desislav TOMOV, Rumyana STOYANOVA, Ivan DECHEV, Yordanka UZUNOVA

**Affiliations:** 1 Department of Urology and General Medicine, Faculty of Medicine, Medical University of Plovdiv, Plovdiv, Bulgaria; 2 Department of Bioorganic Chemistry, Faculty of Pharmacy, Medical University of Plovdiv, Plovdiv, Bulgaria; 3 Department of Health Management and Health Economics, Faculty of Public Health, Medical University of Plovdiv, Plovdiv, Bulgaria; 4 Urology Clinic, “St. George” Hospital, Plovdiv, Bulgaria; 5 Research institute, Medical University of Plovdiv, Plovdiv, Bulgaria

**Keywords:** Prostate cancer, oncometabolites, biomarkers, liquid chromatography with tandem mass spectrometry

## Abstract

**Background/aim:**

Oncometabolites provide a new approach towards the diagnostics and prognosis of the clinical progress of prostate cancer (PCa). This study is about the diagnostic and predictive value of a panel of urinary oncometabolites (ethanolamine, kynurenine, β-alanine, α-alanine, leucine, isoleucine, γ-aminobutyric acid, and sarcosine) and correlation with prostate-specific antigen (PSA) and Gleason score in patients diagnosed with prostate cancer.

**Materials and methods:**

The participants in this cross-sectional study were divided into PCa group (101 patients who matched the including criteria, average age 71) and control group (52 individuals, with no evidence of malignancy, without oncological and other chronic diseases, and without prostate gland pathology, average age 40). The criteria to be included in the PCa group were as follows: i) being diagnosed with prostate cancer, based on digital rectal examination (DRE), prostate ultrasound investigation, or biopsy; ii) not being subjected to a surgical or any other treatment; iii) not having any other concomitant oncological diseases, renal failure, diabetes mellitus. The urinary concentration of the selected metabolites was established through high-performance liquid chromatography with tandem mass spectrometry detection (HPLC-MS/MS).

**Results:**

The comparison of both groups established a significantly different elevated concentration of ethanolamine, sarcosine and kynurenine, and a significantly different decreased concentration of β-alanine and isoleucine in PCa group. No changes of the values were detected in the PCa group with PSA levels below and above 10 ng/mL and Gleason score below and above 6 (p > 0.05). To test whether combination of several variables is more powerful in discriminating between PCa and control group multiple logistic regression analysis was performed. A model including ethanolamine, sarcosine, kynurenine, β-alanine, and isoleucine demonstrated negative predictive power (NPP) 76.2% and positive predictive power (PPP) 81.8%.

**Conclusion:**

Urinary concentrations of ethanolamine, sarcosine, kynurenine, β-alanine, and isoleucine in PCa group differ significantly from that of control group. New expanded population studies are needed to discuss our results.

## 1. Introduction

Prostate cancer (PCa) falls within the top 10 of malignant diseases on a global scale, and it is the second most diagnosed cancer in men; it ranks the fifth among the causes of cancer-related deaths in men worldwide [1, 2]. The growing body of evidence suggests that a great part of the patients with a high-risk disease are not treated, which leads to a further and more expensive therapy for the more advanced or metastatic forms.

The PCa morbidity rate is expected to continue to rise due to demographic changes, but the improved approach for treating the disease would benefit the results while lowering the therapy costs at the same time. Nonspecific or missing symptoms often lead to a delayed diagnosis when treatment is already ineffective, and, therefore, the development of efficient and specific methods for screening and early detection of PCa is extremely important [3].

The symptoms of a locally advanced or metastatic PCa include nonspecific symptoms of the lower urinary tract, which may be associated with benign prostate hyperplasia as well. To this date, digital rectal examination (DRE) and prostate specific antigen (PSA) are the most commonly used methods for early detection, but they cannot predict the clinical progression. Since PSA has a low specificity and is associated with a high number of false-positive results, each induration and nodule formation of the prostate gland, as well as high PSA values, suggest a further assessment through biopsy or imaging diagnostics [3].

For men diagnosed with PCa, there are various algorithms and nomogrames, which are able to predict the probable clinical outcome based on tumor pathology and PSA levels. Even though these methods work relatively well, there is a significant difference in the outcome for males in the groups with low- and high-risk spectrum. On the one hand, 30–50% of the males, identified as low-risk, will need treatment, while for the high-risk patients with Gleason score 8–10, the PCa mortality within a 15-year range could be lower than 40% [4, 5]. These limitations of the current approaches have led to an increased focus on molecular biomarkers with the aim of improving the PCa detection and individual prognosis stratification or the risk of progression. Individual metabolic characteristics of the prostate gland have been known for a long time, but the compilation of a more extensive metabolomic profile of PCa is still at a very early, barely inceptive stage. During the last decade, metabolomics has established itself as a new and promising method associated with PCa biomarkers detection. Metabolomic studies could encompass a specific analysis of a single or a small group of metabolites, linked to a specific metabolic pathway. These metabolites could be an indicator for separate functional disorders in the metabolic pathways, affected by various pathological conditions, including those of PCa patients as well [6,7]

Numerous studies have reported the identification of potential biomarkers, capable of differentiating between a benign and malignant prostate tissue. Fernández-Peralbo et al., [8], have conducted a nontargeted metabolomics urinary analysis of 62 patients with clinically significant PCa and 42 healthy individuals, with both groups confirmed via biopsies. Twenty-eight significant metabolites have been established and proposals have been made for compiling an analytical model, characterized with 88% sensitivity and 93% specificity. Sreekumar et al., [9] metabolomic study of amino acids in the urine of biopsy-positive and biopsy-negative patients single out sarcosine, a glycine metabolite, as a potential PCa biomarker. The study has been widely discussed and interpreted as a demonstration that urinary sarcosine could be used as a promising biomarker for early detection or prognosis. Currently, the role of sarcosine as a PCa biomarker is still debatable. The studies of Colleselli et al. [10] and Cao et al., [11] support this theory. Other authors reject Sreekumar’s thesis and claim that urinary sarcosine could not be used for diagnosing prostate cancer and identifying aggressive tumors [12]. Other studies have found statistically significant differences in terms of leucine, isoleucine, kynurenine, uracil, and γ-aminobutyric acid (GABA) in patients with PCa and a control group without prostate gland pathology, when analyzing urine and plasma [13]. The most recent findings of tissue and urine metabolites with potential for prostate cancer biomarkers were summarized and discussed in a recently published paper. The review unveiled that the development and progression of PCa are mainly associated with alteration of amino acid metabolism, energy metabolism, and membrane metabolism. In the reviewed papers, not only one compound is outlined as a possible biomarker. Multi-biomarker panel has the advantage to capture more deeply various metabolic dysregulations occurring in cancer development and progression than a single biomarker does [14]. The diagnostic variations, including instrumental, imaging, biochemical, genetic, and immunohistological tests give rise to an increased interest in the discovery and study of new biomarkers in different biological matrices that would improve both the early detection of the disease and would act as predictors for its clinical aggressiveness and the success of the treatment as well.

The aim of this study was to evaluate the diagnostic and predictive value of sarcosine, included in a panel of urinary oncometabolites (alanine, β-alanine, ethanolamine, GABA, kynurenine, leucine, and isoleucine) and their correlation with PSA and Gleason score in patients, diagnosed with prostate cancer.

## 2. Materials and methods

### 2.1. Patients

All the procedures of the study involving human participants were performed in accordance with the ethical standards of the Institutional and National Research Committee and within the 1964 Helsinki Declaration and its later amendments or comparable ethical standards. The study design was approved by the Ethics Committee of the Medical University of Plovdiv, Bulgaria (No 6/23.11.2017). The protocol was conducted in accordance with the Helsinki Declaration, Good Clinical Practice guidelines, and national laws. All the procedures were conducted after obtaining written informed consent.

This cross-sectional study was conducted from January 2019 to July 2020 at the Urology Clinic of St. George University Hospital, Plovdiv, Bulgaria. The criteria for including patients into the prostate cancer group were as follows: i) being diagnosed with prostate cancer, based on digital rectal examination (DRE), prostate ultrasound investigation, biopsy, ii) not being subjected to a surgical or any other treatment, and iii) not having any other concomitant oncological diseases, renal failure, and diabetes mellitus. During this period, 328 men with prostate cancer were examined in the clinic, 148 of which were previously diagnosed with PCA and had received any treatment. These patients were excluded from the study. The prostate cancer of the other 180 patients was diagnosed based on increased PSA levels and first biopsy without any previous blood tests for PSA levels and biopsies. These patients were not subjected to a surgical or any other treatment. Fifty-seven out of 180 patients were not included in the study because of the concomitant oncological or chronic diseases such as renal failure and diabetes mellitus. There were 22 patients who refused to sign in written informed consent for religious or ethical reasons. Thus, the PCa group consists of 101 patients not subjected to a surgical or any other treatment (average age 71 (56–87) years). The control group includes 52 men showing no evidence of malignancy without oncological and other chronic diseases and not having prostate gland pathology (average age 40 (28–57) years). They all have been examined for other urological diseases and also signed written informed consent.

### 2.2. Chromatographic analysis

Patients’ medium portion of the first-morning urine has been collected in a sterile container, centrifuged, frozen at −70 °C, and stored until analysis.

The listed metabolites were analyzed through a high-performance liquid chromatography method with tandem mass spectrometry (HPLC-MS/MS). The detection ranges are as follows: sarcosine 0.004–0.500 mg/L; β-alanine, GABA and isoleucine 0.078–10.00 mg/L; isoleucine 0.156–20.000 mg/L; alanine, kynurenine, and ethanolamine 0.78–100.000 mg/L. A brief description of the method is as follows: On the day of analyses, urine sample was thawed, centrifuged, diluted 2 times, and subjected to derivatization with danzylchloride at pH = 9.5 (0.5 M NaHCO_3_) for 15 min at 25 °C. At the end of the reaction time, 15 μL 10% solution of methanoic acid were added to stop the reaction. The solution was diluted 100-fold and injected in the Dionex /Thermo UltiMate 3000 chromatographic system (Thermo Fischer Scientific, MA USA) equipped with Accucore reversed-phase mass spectrometry (100mm × 2.1mm, 2.6 μm) column (Thermo Fischer Scientific, MA USA). Gradient elution was performed with phase A (0.1% НСООН in water) and phase B (0.1% НСООН in 60% CH_3_CN) with flow rate 0.1 mL/min. Autosampler temperature was 4 °C, and volume of injection was 10 μL. Analytes were detected with Thermo TSQ Quantum Access Max triple quadrupole mass spectrometer operated in positive ion mode using electrospray ionization (ESI) within the following conditions: spray voltage 3500V, evaporation temperature 300 °C, capillary temperature 200 °C, sheath gas (N_2_) pressure 45 units, collision gas (Ar) 1.5 mTorr. Analytes were detected through the transition of the molecular ion to the production at the corresponding retention times. Their concentration was back-calculated by using calibration curves prepared in pooled urine samples. The results were registered and processed with Xcalibur software (Thermo Fischer Scientific, MA USA) in SRM mode.

### 2.3. Statistical analysis

The data were analyzed with descriptive statistics, Kolmogorov–Smirnov normality test and nonparametric test using GraphPadPrism 9.0.0 version (GraphPad Software, 2365 Northside Dr.Suite 560, San Diego, CA 92108, USA). The binary comparison continuous data was analyzed with Mann–Whitney U test. The comparison among three groups (low, middle, and high risk) was done with Kruskal–Wallis test and Dunn’s post hoc test. ROC curve analysis was done for investigating the diagnostic potential of the metabolites with elevated or reduced concentrations with a statistical difference in both groups: sarcosine, β-alanine, ethanolamine, GABA, kynurenine, and isoleucine. To test whether the combination of several variables is more powerful in discriminating between PCa patients and the control group, multiple logistic regression analysis (Hosmer–Lemeshow tets) was performed, and multivariable ROC curves were build. The level of significance of 5 % probability (p < 0.05) was adopted.

## 3. Results

**Figure 1:**
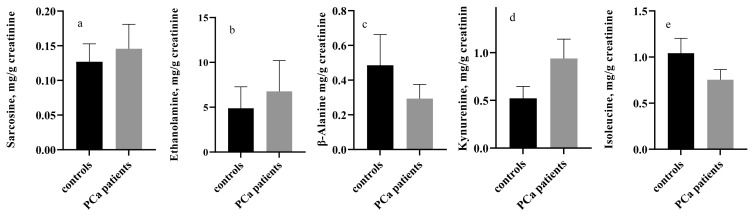
Median values with 95% confidence interval of sarcosine (a), ethanolamine (b), β-alanine (c), kynurenine (d) and isoleucine (e) in the control group and the PCa group.

**Figure 2:**
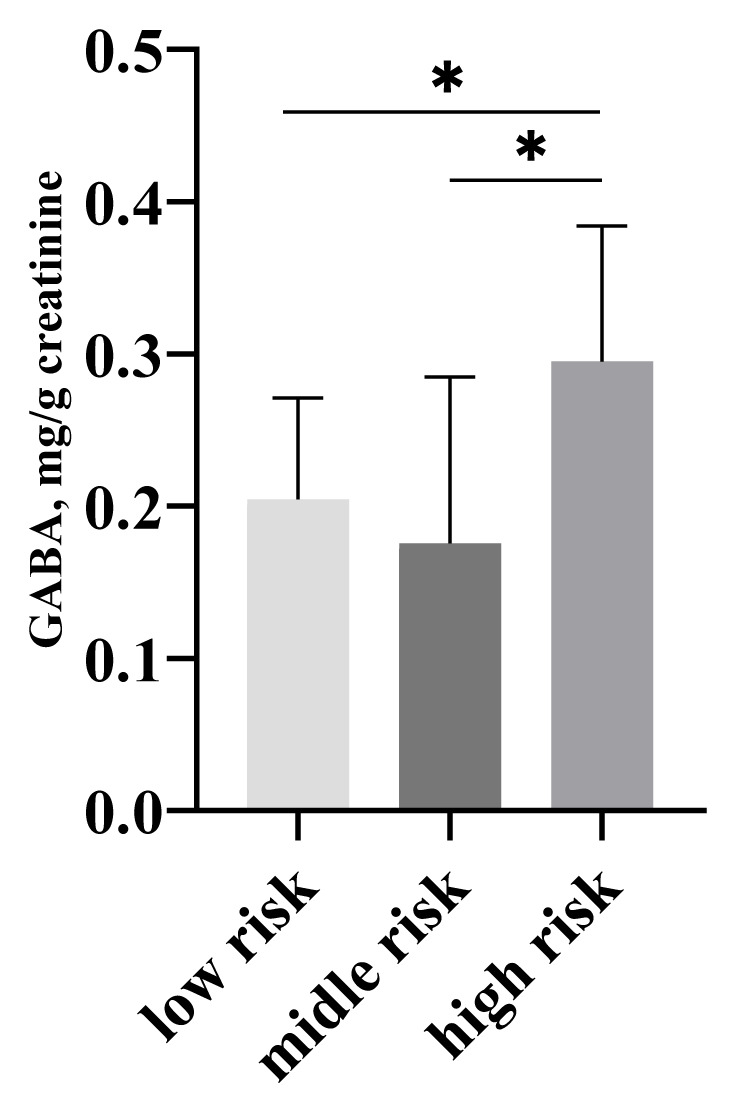
Median values with 95% confidence interval of GABA in patients with high, middle, and low risk of the PCa group. *statistically significant difference between median values.

The group of PCa patients includes 101 cases, histologically verified via biopsy, Gleason score, PSA test and ultrasound examination of the urinary tract system but not subjected to a surgical or any other treatment. The average PSA level was 22.73 ng/mL, and median Gleason score was 7. The concentrations of all metabolites were determined in both PCa (n = 101) and control group (n = 52). Urinary samples from all patients were subjected to a chromatographic analysis. The results were normalized in relation to creatinine, determined by the Jaffe method (15). The comparison of the results has been made with a Mann-Whitney U test and has established a significant difference in ethanolamine (6.77 PCa group vs 4.88 control group, p = 0.044), sarcosine (0.146 PCa group vs 0.105 control group, p = 0.003), kynurenine (0.940 PCa group vs 0.522 control group, p<0.001), β-alanine (0.294 PCa group vs 0.486 control group, p = 0.045) and isoleucine (0.755 PCa group vs 0.952 control group, p = 0.042) (Figure 1). The concentration of metabolites were also compared within the PCa group and the patients were divided into such with Gleason score up to (n = 47) and above 6 (n = 54), and PSA values up to (n = 42) and above 10 ng/mL (n = 59). No changes of the values of the studied metabolites were detected in the group with verified prostate cancer with PSA levels below and above 10 ng/mL (p > 0.05). Statistically significant changes have not been found in the values of the studied metabolites in patients with verified prostate cancer, stratified in terms of Gleason score (GS) below (n = 47) and above 6 (n = 54) (ethanolamine p = 0.627, β-alanine p = 0.278, alanine p = 0.241, GABA p = 0.288, sarcosine p = 0.260, kynurenine p = 0.623, isoleucine p = 0.367 and leucine p = 0.353). For that reason, patients were divided in three subgroups: low risk: PSA<10 ng/mL, GS<7 (n = 38); middle risk: PSA 10–20 ng/mL, GS=7 (n = 36) and high-risk PSA>20 ng/mL, GS>7 (n = 27) according to the EAU recommendations [2]. From all the investigated metabolites only γ-aminobutyric acid (GABA) levels were found to be statistically different elevated in the high-risk group compared to both middle and low risk groups: high risk vs low risk p = 0.045; high risk vs. middle risk p = 0.025 (Figure 2).

For the metabolites with statistically significant difference in concentrations ROC analysis was performed. The AUC varies between 0.60 and 0.72 (Table 1). Results revealed the kynurenine presented the highest significant diagnostic value (AUC: 0.72, sensitivity 70%, specificity 65.4% p<0.001), followed by sarcosine (AUC: 0.64, sensitivity 71.3%, specificity 50% p = 0.003).

Ability of a panel of metabolites to discriminate the healthy individuals from PCa patients was assessed with multiple logistic regression analyses. The multivariable ROC curve’s area under curve (AUC) increases with the number of included variables (Table 2). The negative predicted power (NPP) and positive predicted power (PPP) of models are as follows: kynurenine/isoleucine model has NPP 68.3% and PPP 78.4%; ethanolamine/kynurenine/isoleucine model-NPP 71.4% and PPP 80%; kynurenine/β-alanine/isoleucine model-NPP 72.7% and PPP 80%; sarcosine/kynurenine/β-alanine/isoleucine model-NPP 73.7% and PPP 81.5%; ethanolamine/sarcosine/kynurenine/β-alanine/isoleucine model-NPP 76.2% and PPP 81.8%.

The Spearman correlation between oncometabolites and PSA levels was studied only in PCa group. A very weak negative correlation was found between etanolamine, GABA and kynureniene and PSA in group with PSA<10 ng/mL- r = −0.15, r = −0.26 and r = −0.11 respectively. Only GABA shows very weak correlation with PSA in the group with PSA ≥ 10 mg/mL r = 0.1.

## 4. Discussion

The characterization of metabolites, derived through specific processes in cancer cells or as a result of metabolic pathway disorders, is a strategy for the discovery of new methods for early detection of pathological changes in the prostate gland and the study of prostate cancer progression. Many studies were conducted in an attempt to evaluate the role of amino acids as metabolites with potential as prostate cancer biomarkers. Prostate cancer was studied using metabolomics profiling in order to get insight into the entire measurable metabolome. There are a few studies that are focused on targeted metabolites [16, 17]. Out of numerous analytes studied in urine, plasma, and prostate tissue via biopsy, ethanolamine, sarcosine, β-alanine, alanine, kynurenine, GABA, isoleucine, and leucine are found to be suitable for compiling a panel for prostate pathological changes, so the present study focuses on these 8 metabolites.

Since the number of samples analyzed in our study is limited (101 prostate cancer patients and 52 controls), it should be considered as a pilot study. The presented research gives the opportunity to see the outcomes of previous studies on amino acid profile changes in prostate gland cancer [14, 18, 19] and also provides new data on the levels of some other amino acids and metabolites, which are not so well examined in prostate cancer biomarker investigations such as ethanolamine, kynurenine, isoleucine, and β-alanine. However, only sarcosine is currently studied for its potential role in prostate cancer progression.

Urine has been selected as biological matrix due to the noninvasive sampling method, easy further processing, and high metabolite content. Urine samples from 52 controls and 101 prostate cancer patients were analyzed with HPLC-MS/MS method. The obtained results proved that prostate cancer causes changes in five of the studied metabolites. Significantly higher median values are observed for ethanolamine (6.773, p = 0.044), sarcosine (0.146, p = 0.003) and kynurenine (0.940, p < 0.001) in PCa group compared to the controls, while β-alanine and isoleucine show significantly lower median values compared to the controls (0.294, p = 0.048 and 0.755, p = 0.042 respectively), (Figure 1).

Sarcosine, also known as N-methylglycine is an intermediate product of the amino acid glycine synthesized by glycine-N-methyltransferase. The predictive role of sarcosine was discussed for the first time in the study of Sreekumar et al., [9]. The authors focus on the potential of the molecule panel within the sarcosine metabolic pathway to provide biomarkers for understanding the biology of PCa. The study includes independent metabolomic profiling through liquid chromatography/gas chromatography mass-spectrometry, detecting six metabolites, the levels of which were higher in tumor progression when compared to benign prostate tissue, adjacent to a tumor tissue, and in comparison to a localized PCa and metastatic PCa. Those metabolites are sarcosine, uracil, kynurenine, L-leucine, proline, and glycerol-3-phosphate. Among them, sarcosine has shown the most prominent differences. The same authors found that sarcosine is a potential mediator of PCa progress. The metabolomic profile of the progressing PCa identifies the gradually increasing sarcosine level in metastatic PCa, as well as a moderate but significant increase in the level of the metabolite in urine in verified PCa. Other authors reported different findings of sarcosine concentration in urine and do not confirm its role in early detection and clinical progress of prostate cancer [10]. Our results confirm significantly different sarcosine concentration in the urine of both groups (Figure 1a).

Ethanolamine is a degradation product of phospholipid membranes, and it is involved of serine metabolism. It is not an amino acid, but a primary amine and a primary alcohol. Its presence in urine can be indicative for significant processes in the human body like cellular proliferation apoptosis, and enzyme activity. The concentration of ethanolamine is significantly higher in the urine of PCa patients (median value 6.773, p = 0.044) (Figure 1b), while Derezinski et al. [18] and Swanson et al. [19] reported lower levels of its concentration in PCa patients vs. controls. The elevated concentration of ethanolamine might be associated with extreme cellular proliferation of tumor cells and the aggressiveness of cancer.

In the current study, α-alanine and β-alanine were included as isomers of sarcosine. The objective was to separate them from sarcosine in order to determine their precise concentration in the biological matrix. This was cited as a drawback in previous studies in which an elevated concentration of sarcosine might have been due to simultaneous elution of sarcosine and its isomers from the chromatographic column [20]. We were able to successfully separate the three isomers and established a significantly lower concentration of β-alanine in patients with PCa (Figure 1c). There is limited data on the role of β-alanine in the development and progression of PCa, which needs further elucidation. A study conducted by Derizinski et al. [18] did not detect significantly different changes in the levels of β-alanine in urine and serum of prostate cancer patients compared to the control group of healthy individuals.

Compared to the control group, a significantly higher kynurenine concentration was found in the PCa group (Figure 1d). Metabolites of the kynurenine pathway have been reported to increase during the development of prostate cancer [9, 16, 17]. The presented data are in accordance with a previous study by Sreecumar at al. [9] who found increased kynurenine concentration in urine. Kynurenic acid (metabolite of the kynurenine pathway) was also found to increase in the urinary samples of 32 patients with PCa before radical prostatectomy and the samples of 101 patients with increased PSA values before an ultrasound-guided biopsy [17]. However, the analysis showed that sarcosine and kynurenic acid do not have any diagnostic value. Univariate ROC curve analyses of our results revealed that the kynurenine presented the highest significant diagnostic value (AUC: 0.719, p < 0.0001), followed by sarcosine (AUC: 0.645, p = 0.003) (Table 1.).

Isoleucine is a part of the group of branched–chain amino acids together with leucine and valine. Our findings on significantly lower concentrations of isoleucine in PCa group are consistent with the results of Derezinski et al. [18], who found a decreased urine and serum level of isoleucine in the case of prostate cancer (Figure 1e). The diagnostic ability of all five metabolites is higher than the adopted level of significance but AUC is still low than 0.750.

In the group of prostate cancer additional analyses were performed in order to establish whether there were statistically significant changes in the concentrations of studied metabolites. PSA values from 4 to 10 ng/mL are included in the so-called grey area, within which the causes of the increase could be due not only to malignant diseases but to benign changes and prostatitis as well. In a great number of cases, PSA values up to 10 ng/mL in patients with verified PCa is associated with an earlier stage of the disease, better therapeutic options, and a more favorable outcome, while values above 10 ng/mL are associated with more advanced processes and worse prognosis. Therefore, the joint study of both PSA values and metabolite concentrations in patients with verified prostate cancer would give a better view on the malignant potential and prognosis for the tumor. The weak correlation between metabolites and PSA levels indicates that its level may not strongly affect metabolism of amino acids.

Gleason score is used as a method for histological differentiation of tumors, clinical course of the disease and prognostic group. Men with higher Gleason score fall in a group with poorer prognosis, their carcinomas are less differentiated, more aggressive, and with worse outcome. Elevated γ-aminobutyric acid concentration can be associated with worse prognosis for patients with GS>7 and PSA>20 ng/mL and higher risk of organ metastases.

The utility of panel of metabolites to discriminate the samples with high accuracy was demonstrated by Derezinski et al., [18]. The panel including 5 metabolites showed 78% predictive accuracy. The multiple logistic regression models including two, three, four, and five metabolites were analyzed, and multivariable ROC curves were built. The models presented in Table 2 demonstrate the highest AUC and increased NPP and PPP. Kynurenine and isoleucine are present in all models whereas sarcosine is present in only two of them. These findings allow proposing future directions of research – special attention should be paid to kynurenine and isoleucine. The example of ethanolamine, β-alanine, and sarcosine indicated that nonproteinogenic amino acids and other metabolites can contribute to prostate cancer pathogenesis and improvement of its detection. Metabolomic analyses have outlined other amino acids that have statistically different concentrations in healthy individuals and prostate cancer patients which opened the gate for future analyses of other panels of metabolites in order to find the best model for detection, and diagnostic and prognostic of the disease.

One of the limitations of our study is the absence of demographic data. On the other hand, the control group was recruited from individuals presenting for other etiologies and these patients are much younger than patients in the PCa group. In addition, some of the selected metabolites are still in the early stages of investigation as potential biomarkers and our results need to be verified in larger studies and other population groups.

The present study focuses on metabolites with an established role in the pathogenesis of PCa cancer (sarcosine) as well as some amines and amino acids, which have been recently outlined as possible biomarkers. To the best of our knowledge, this panel has not been studied by the other authors. It outlined several metabolites (ethanolamine, β-alanine, sarcosine, isoleucine, GABA, kynurenine) with significant changes in their concentrations which need further investigation. The proposed model for discriminating PCa and control group, which includes ethanolamine, β-alanine, sarcosine, kynurenine, and isoleucine could be used for urinary metabolite determination for possible early cancer detection and these five metabolites could be used as biomarkers.

## Figures and Tables

**Table 1:** Area under the curve (AUC), 95% confidence intervals (CI) and p values for ethanolamine, sarcosine, kynurenine, b-alanine, and isoleucine.

Metabolites	AUC	95% CI	Cut-off values	Sensitivity, %	Specificity,%	p
Ethanolamine	0.599	0.507–0.690	5.552	58	56	0.044
Sarcosine	0.645	0.552–0.737	0.100	71.3	50	0.003
Kynurenine	0.719	0.638–0.800	0.648	70	65.4	<0.001
β-Alanine	0.597	0.497–0.696	0.460	74.3	54	0.049
Isoleucine	0.600	0.503–0.697	0.914	66.3	56	0.042

Cut-off values are given in mg/g creatinine.

**Table 2:** Area under the curve (AUC), 95% confidence interval (95% CI), p values.

Model	AUC	95% CI	p
Kynurenine+Isoleucine	0.802	0.730–0.873	<0.0001
Ethanolamine+Kynurenine+Isoleucine	0.813	0.744–0.883	<0.0001
Kynurenine+β-Alanine+Isoleucine	0.832	0.767–0.898	<0.0001
Sarcosine+Kynurenine+β-Alanine+Isoleucine	0.848	0.785–0.912	<0.0001
Ethanolamine+Sarcosine+Kynurenine+β-Alanine+Isoleucine	0.860	0.799–0.921	<0.0001
